# Anthropometric Measures and Risk of Cardiovascular Disease: Is there an Opportunity for Non-Traditional Anthropometric Assessment? A Review

**DOI:** 10.31083/j.rcm2312414

**Published:** 2022-12-20

**Authors:** Aurora Carrión-Martínez, Benjamin J R Buckley, Esteban Orenes-Piñero, Francisco Marín, Gregory Y. H Lip, José Miguel Rivera-Caravaca

**Affiliations:** ^1^Department of Cardiology, Hospital Clínico Universitario Virgen de la Arrixaca, University of Murcia, Instituto Murciano de Investigación Biosanitaria (IMIB-Arrixaca), CIBERCV, 30120 Murcia, Spain; ^2^Liverpool Centre for Cardiovascular Science at University of Liverpool, Liverpool John Moores University and Liverpool Heart & Chest Hospital, L14 3PE Liverpool, UK; ^3^Cardiovascular and Metabolic Medicine, Institute of Life Course and Medical Sciences, University of Liverpool, L69 3BX Liverpool, UK; ^4^Department of Biochemistry and Molecular Biology-A, University of Murcia, Instituto Murciano de Investigación Biosanitaria (IMIB-Arrixaca), CIBERCV, 30120 Murcia, Spain; ^5^Department of Clinical Medicine, Aalborg Thrombosis Research Unit, Aalborg University, L69 3BX Aalborg, Denmark

**Keywords:** myocardial infarction, stroke, diabetes, hypertension, kinanthropometry, body weights and measures

## Abstract

**Background:**

Several anthropometric measurements are used to assess cardiovascular risk and progress during clinical treatment. Most commonly used anthropometric measurements include total body weight and body mass index (BMI), with several other simple anthropometric measures typically underused in clinical practice. Herein, we review the evidence on the relationship between different anthropometric measurements and cardiovascular risk in patients with and without cardiovascular disease (CVD).

**Methods:**

Data for this review were identified by searches in PubMed, the Web of Science, Google Scholar, and references from relevant articles by using appropriate and related terms. The last search was performed on June 22, 2022. Articles published in English and Spanish were reviewed and included, if appropriate. We included studies detailing the relationship between skinfolds thickness, waist-to-hip ratio (WHR) and Conicity index with cardiovascular risk in adults with/without CVD.

**Results:**

In patients from the general population, elevated subscapular and triceps skinfolds showed a positive relationship with the development of hypertension, diabetes mellitus, hypercholesterolemia, cardiovascular mortality, and all-cause mortality. A higher subscapular skinfold was also associated with increased risk of coronary artery disease and stroke. A higher WHR, as well as other less common anthropometric measurements such as the Conicity index, was associated with an increased risk of myocardial infarction, incident CVD, major adverse cardiovascular events, and mortality in both patients with and without previous CVD.

**Conclusions:**

Non-traditional anthropometric measurements including skinfolds and WHR seem to improve the prediction of cardiovascular risk in the general population, and recurrent events in patients with previous CVD. Use of additional anthropometric techniques according to an objective and standardized method, may aid cardiovascular risk stratification in patients from the general population and the evaluation of therapeutic interventions for patients with CVD.

## 1. Introduction

Cardiovascular disease (CVD) is the most common cause of morbidity and mortality worldwide, with ischemic heart disease and stroke as the leading causes of CVD-related deaths [1, 2].

Comprehensive treatment and prevention of CVD should include adherence to a healthy diet, a healthy body composition, and regular physical exercise (150 minutes of moderate to vigorous intensity). It is well known that an unhealthy lifestyle associates with cardiovascular risk factors, and can contribute to excess accumulation of (visceral) fat and subsequently lead to atherosclerotic processes [3, 4, 5]. In fact, body composition and particularly the presence of elevated body fat, are closely related to the development and onset of CVD [6].

For this reason, anthropometric measurements are frequently used to assess the clinical evolution of patients, and to control cardiovascular risk factors. However, traditional anthropometric measurements focus mainly on total body weight and body mass index (BMI), underutilizing other potentially useful parameters that could improve risk prediction and risk factor monitoring. Other anthropometric measures such as waist-to-hip ratio (WHR) and fat distribution (according to skinfold thickness) have been related to cardiovascular risk factors [7].

In the present study, we have performed a review to summarise the relationship of non-classic anthropometric measures, CVD and associated risk factors, and determine the potential usefulness of including them in routine clinical practice.

## 2. Methods

Eligible studies included patients with CVD, particularly coronary artery disease (CAD); and participants without CVD at baseline but who developed CVD, associated risk factors (hypertension, diabetes mellitus, or hypercholesterolemia), or major adverse events (stroke, all-cause death, CV-related death) during follow-up. All included studies investigated the relationship of the above outcomes with anthropometric variables including traditional parameters (body weight and BMI) as well as other less frequently used measures including WHR, skinfold thickness, and the Conicity index. Although there are numerous variations of skinfold thickness procedures, we focussed on subscapular skinfold (SSF) and triceps skinfold (TSF), as these are the most commonly researched. The Conicity index (according to the following formula: waist circumference (m) / [0.109 ×$\sqrt{\text{(body weight (kg) / height (m))}}$], where 0.109 is a constant) was also included because of its particular interest for the aim of this review.

Included studies for this review were identified by searches of PubMed, the Web of Science, Google Scholar, and references from relevant articles. Searches included the following terms which were combined with Boolean operators “AND”, “OR”, and “NOT”: “myocardial infarction”, “coronary artery disease”, “acute coronary syndrome”, “cardiac rehabilitation”, “hypertension”, “diabetes”, “stroke”, “kinanthropometry”, “subscapular skinfold”, “triceps skinfold”, “Conicity index”, “body mass index”, “waist circumference”, and “waist-to-hip ratio” without filters by year (last search in June 22, 2022). Articles published in English and Spanish were reviewed and included, if appropriate.

## 3. Anthropometric Measurements Less Frequently Used in Patients with Cardiovascular Disease

It is well known that obesity increases the risk for CVD [8]. Classically, the most widely used anthropometric parameters in clinical practice have been height, body weight and BMI, as they do not require specialist training. However, there are several more advanced anthropometric measures and indices based on the measurement of skinfolds, perimeters, lengths and diameters [9], that may have prognostic utility.

### 3.1 Subscapular and Triceps Skinfolds Thickness

The usefulness of SSF as a measure for estimating cardiovascular risk is well known. In three classic studies, central obesity estimated by SSF, was shown to be a significant predictor of CAD following >10 years follow-up, which was independent of BMI [10, 11, 12]. Later, in a long-term study in 10,582 Japanese patients with type 2 diabetes mellitus (T2DM), TSF and SSF were significantly higher in patients with impaired fasting glucose levels (*p *< 0.001 and *p* = 0.001, respectively). Compared to patients with normal glucose levels, non-hypertensive diabetic subjects with high TSF had a 3.6-fold higher relative risk (RR) for non-embolic ischemic stroke (RR 3.6; 95% confidence interval [CI] 1.7–7.4), and non-hypertensive diabetic subjects with elevated SSF had a 4.9-fold higher risk (RR 4.9; 95% CI 2.5–9.5) [13]. A separate study, including more than 9000 participants without CVD followed for 23 years, showed that mortality rates associated with fatal stroke or CVD increased as SSF did. The SSF was found to be independently associated with mortality from CAD and stroke, and subjects with a SSF in the upper quartile had a significantly higher risk compared to the lowest quartile (for CAD-related mortality: HR 1.06; 95% CI 1.00–1.13; for stroke-related mortality: HR 1.12; 95% CI 1.01–1.25) [14].

One study followed participants without hypertension or diabetes for ten years in two communities (n = 2422 and 3195), and investigated the relationship between the occurrence of these diseases with anthropometric measurements. The significant predictors of hypertension were BMI and WHR, and for diabetes were BMI and SSF in both sexes and in both communities (except in men from one of the communities) [15]. Another study carried out on 8892 Asian participants between 20 and 60 years of age compared the performance of six obesity indices as tools to identify adults with cardiovascular risk factors. The authors found that less commonly used measures in the healthcare setting, such as the sum of the TSF to SSF, showed a good correlation with BMI, and a moderate predictive ability for diabetes, hypercholesterolemia and hypertension (all with a c-index >0.68) [16].

During almost 30 years of follow-up, another study investigated whether skinfold measurements were associated with mortality regardless of variation in BMI in 870 apparently healthy adult men. In the univariate analysis, BMI was associated with all-cause mortality, cancer-related mortality, arteriovascular-related mortality, and other mortality. The SSF was associated with all-cause mortality and arteriovascular-related mortality. However, in multivariate analyses, the SSF showed no association, but a low iliac skinfold emerged as a strong independent risk factor for all-cause mortality, arteriovascular-related mortality and infectious mortality [17].

Another study tried to determine the association between excess body fat, assessed by skinfold thickness, and the incidence of T2DM and hypertension. Bicipital skinfold and SSF were associated with 2.8 and 6.4-fold risk of developing T2DM, while overall and subscapular fat obesity were associated with a 2.9 and 2.4-fold risk of developing hypertension [18].

Finally, a recent study investigated the associations between SSF and TSF with all-cause, cardiovascular, and cerebrovascular mortality in a large American cohort, demonstrating an inverse association of both parameters with all-cause and cardiovascular mortality [19, 20]. Table 1 (Ref. [10, 11, 12, 13, 14, 15, 16, 17, 18, 19, 20]) summarizes the main results of the above studies.

**Table 1.** **Studies included in the review about subscapular skinfold and triceps skinfold**.

Study	Population	Sample size	Follow-up	Main outcomes
Donahue *et al*. [10]	Patients free of previous CVD	7692	12 years	Increased incidence of CAD in patients with the highest tertile of the SSF (80 patients per 1000, *p *< 0.001).
				Increased risk of developing CAD with higher SSF:
				∙ RR 2.2 (95% CI 1.8–2.8)
				[tertile 3 *vs*. tertile 1, adjusted by age].
				∙ RR 1.5 (95% CI 1.1–2.1)
				[tertile 3 *vs*. tertile 1, adjusted by risk factors].
Kannel *et al*. [12]	General population	5209	24 years	Increased risk of developing CVD with higher SSF (quintile 5 *vs*. quintile 1).
				∙ CAD: RR 1.8 (*p *< 0.001) (males); RR 1.8 (*p *< 0.001) (females).
				∙ Stroke: RR 1.7 (*p *< 0.01) (females).
				∙ Any CVD: RR 1.4 (*p *< 0.001) (males); RR 1.7 (*p *< 0.001) (females).
				∙ CAD-related mortality: RR 1.4 (*p *< 0.001) (males); RR 2.0 (*p *< 0.001) (females).
				∙ Cardiovascular mortality: RR 1.4 (*p *< 0.001) (males); RR 1.5 (*p *< 0.001) (females).
Yarnell *et al*. [11]	Patients (males) free of previous CVD	2512	14 years	Increased risk of developing CAD with higher SSF:
				∙ OR 1.23 (95% CI 1.04–1.45)
				[per standard deviation of increase, adjusted for BMI].
				∙ RR 1.90 (95% CI 1.30–2.80)
				[quintile 5 *vs*. quintile 1, adjusted for age, smoking habit and social class].
Iso *et al*. [13]	General population	10,582	17 years	Increased risk of non-embolic ischemic stroke in non-hypertensive diabetic subjects with higher SSF (RR 4.9; 95% CI 2.5–9.5) and TSF (RR 3.6; 95% CI 1.7–7.4).
Tane *et al*. [14]	Patients (males) free of previous CVD	9151	23 years	Higher overall mortality rate from CAD or stroke in the fourth quartile of the SSF.
				Increased risk of mortality from CAD with higher SSF:
				∙ HR 1.13 (95% CI 1.06–1.20) [adjusted for age].
				∙ HR 1.06 (95% CI 1.00–1.13)
				[adjusted for age and hypertension].
				Increased risk of mortality from stroke with higher SSF:
				∙ HR 1.12 (1.01–1.25) [adjusted for age].
Chei *et al*. [15]	General population from two communities	5617	10 years	Increased risk of developing hypertension in females from one of the communities with the highest SSF:
				∙ OR 1.60 (95% CI 1.04–2.46) [tertile 3 *vs*. tertile 1].
				Increased risk of developing diabetes in females from both communities with higher SSF:
				∙ OR 2.06 (95% CI 1.05–4.04) [tertile 3 *vs*. tertile 1].
				∙ OR 3.58 (95% CI 1.33–9.64) [tertile 3 *vs*. tertile 1].
Patel *et al*. [16]	General population	8892	N/A	Moderate predictive ability of the sum of the TSF to SSF in patients between 20 and 60 years of age for:
				∙ Hypercholesterolemia (c-index = 0.617 [males]; 0.689 [females]).
				∙ Diabetes (c-index = 0.764 [males]; 0.774 [females]).
				∙ Hypertension (c-index = 0.693 [males]; 0.768 [females]).
Loh *et al*. [17]	Patients (males) free of previous CVD	870	27.7 years	Increased risk of mortality with the lowest iliac skinfold:
				∙ All-cause mortality: HR 0.77 (95% CI 0.66–0.90).
				∙ Arteriovascular mortality: HR 0.75 (95% CI 0.58–0.97).
				∙ Infection mortality: HR 0.63 (95% CI 0.42–0.94).
Ruiz-Alejos *et al*. [18]	General population	988	7.6 years	Increased risk of diabetes in patients with higher SSF:
				RR 5.04 (95% CI 1.85–13.73).
				Increased risk of hypertension in patients with higher SSF: RR 2.15 (95% CI 1.30–3.55).
				Both models adjusted for age, sex, education, assets index, smoking, excessive alcohol consumption, population group, level of physical activity and BMI.
Liu *et al*. [19]	General population	16,402	11.81 years	Lower risk for all-cause and cardiovascular mortality in participants in the highest quartile of SSF.
				∙ All-cause mortality: HR 0.71 (95% CI 0.57–0.89) [quartile 4 *vs*. quartile 1].
				∙ Cardiovascular mortality: HR 0.44 (95% CI 0.23–0.83) [quartile 4 *vs*. quartile 1].
				Both models adjusted for age, gender, race, education level, marital status, smoking, alcohol consumption, BMI, systolic blood pressure, estimated glomerular filtration rate, high-density lipoprotein cholesterol, total cholesterol, C-reactive protein, comorbidities, and medication use.
Li *et al*. [20]	General population	62,160	119 months	Lower risk for all-cause and cardiovascular mortality in participants in the highest quartile of TSF.
				∙ All-cause mortality: HR 0.64 (95% CI 0.54–0.76) [quartile 4 *vs*. quartile 1].
				∙ Cardiovascular mortality: HR 0.54 (95% CI 0.36–0.79) [quartile 4 *vs*. quartile 1].
				Both models adjusted for age, gender, race, waist circumference, education level, marital status, smoking, BMI, estimated glomerular filtration rate, high-density lipoprotein cholesterol, total cholesterol, and comorbidities.

RR, relative risk; OR, odds ratio; HR, hazard ratio; CI, confidence interval; CVD, cardiovascular disease; SSF, subscapular skinfold; TSF, triceps skinfold; CAD, coronary artery disease; BMI, body mass index.

### 3.2 Waist-to-Hip Ratio

In a case-control study carried out by Yusuf *et al*. [21] including 27,098 patients, the risk of acute myocardial infarction (AMI) significantly increased with higher WHR, both when WHR was evaluated as continuous variable and as quintiles. Interestingly, in 3734 patients with Non-ST Segment Elevation Myocardial Infarction (NSTEMI), the highest mortality rate occurred in patients with the lowest BMI but the highest WHR [22].

In another of study, Myint *et al*. [23] observed a higher risk of developing CVD in the general population of both sexes, as well as a higher risk of mortality in women, with higher WHR. Even in patients who already suffer from CAD, previous evidence showed that as WHR increased, rates of major adverse cardiovascular events (MACE) and mortality were also higher, again with a stronger association in women [24].

In Norway, a study with more than 140,000 patients without CVD showed that the population attributable fraction of AMI associated with WHR in the upper two quintiles (i.e., the fraction of all cases of AMI that was attributable to WHR) was 26.1% (95% CI 14.6–36.1) for middle-aged women (<60 years) and 9.3% (95% CI 3.0–15.1) for similarly aged men, after adjusting for BMI and conventional cardiovascular risk factors. However, these observations were not confirmed in patients >60 years of age [25].

Finally, in a study by Medina-Inojosa *et al*. [26], the risk of MACE was significantly and positively associated with a higher WHR, which remained after adjusting for BMI. Table 2 (Ref. [21, 22, 23, 24, 25, 26]) summarizes the results of the studies investigatingWHR and cardiovascular risk included in this review.

**Table 2.** **Studies included in the review about waist-to-hip ratio**.

Study	Population	Sample size	Follow-up	Main outcomes
Yusuf *et al*. [21]	General population (controls) and patients with a first episode of MI (cases)	27,098	N/A	Increased risk of MI with higher WHR:
				∙ OR 1.37 (95% CI 1.33–1.40)
				[for each standard deviation, adjusted for age, sex, region, BMI and height].
				∙ OR 1.33 (95% CI 1.16–1.53)
				[quintile 5 *vs*. quintile 1, adjusted for age, sex, region, BMI, height, smoking, apolipoproteins, hypertension, diabetes, diet, physical activity, alcohol, and psychosocial variables].
Lee *et al*. [22]	Patients with STEMI	3734	199 days	Increased risk of mortality in patients with the highest WHR (>1.0 in males and >0.95 in females):
				∙ HR 5.57 (95% CI 1.53–12.29)
				Adjusted for age, sex, Killip, blood pressure, left ventricular ejection fraction, MI or previous angina, heart failure, hypertension, diabetes, smoking, levels of lipids, stroke, peripheral artery disease, previous and after discharge medications, reperfusion therapies and angiographic findings].
Myint *et al*. [23]	General population	15,062	11.7 years	Increased risk of mortality with higher WHR:
				∙ HR 1.42 (95% CI 1.14–1.78) (in females).
				Increased risk of developing CVD with higher WHR:
				∙ Males: HR 1.17 (95% CI 1.01–1.36).
				∙ Females: HR 1.36 (95% CI 1.16–1.58).
				All models adjusted for age, smoking, alcohol consumption, physical activity, social class, education, blood pressure, cholesterol, diabetes, stroke, MI, cancer, % of body fat and BMI.
Lee *et al*. [24]	Patients with STEMI	2995	1 year	Increased risk of MACE (any of the following: all-cause death, MI, coronary revascularization) with higher WHR:
				∙ OR 1.87 (95% CI 1.29–2.71) [tertile 3 *vs*. tertile 1, adjusted for BMI].
Egeland *et al*. [25]	General population	140,790	11.5 years	Increased risk of MI with higher WHR:
				∙ Males <60 years: HR 1.22 (95% CI 1.07–1.40).
				∙ Females <60 years: HR 1.76 (95% CI 1.37–2.25).
				Both models for the two highest quintiles and adjusted for age, smoking, BMI, systolic blood pressure, diabetes, and total cholesterol-HDL ratio.
Medina-Inojosa *et al*. [26]	Patients with CAD	1529	5.7 years	Increased risk of MACE (any of the following: ACS, coronary revascularization, ventricular arrhythmias, stroke or all-cause death) in females with higher WHR:
				∙ HR 1.75 (95% CI 1.07–2.87) [tertile 3 *vs*. tertile 1, adjusted for BMI].

OR, odds ratio; HR, hazard ratio; CI, confidence interval; MI, myocardial infarction; CVD, cardiovascular disease; MACE, major adverse cardiovascular events; WHR, waist-to-hip ratio; CAD, coronary artery disease; ACS, acute coronary syndrome; STEMI, ST-segment elevation myocardial infarction; BMI, body mass index.

### 3.3 Are There Other Anthropometric Measurements Useful in Cardiovascular Disease?

Apart from WHR and skinfold thickness, we considered the inclusion of other anthropometric measurements or indices that could be interesting for the assessment of patients with CVD.

A recent study including 1488 elderly people (mean age of 69.7 ± 7.30 years) from the general population aimed to compare the predictive ability of different anthropometric parameters for metabolic syndrome (MetS). The authors found that waist-to-standing height ratio (WHtR) presented the highest performance for predicting MetS (c-index = 0.786, 95% CI 0.76–0.81) [27].

In a study that included more than 50,000 women between 40 and 70 years of age with no history of CAD, stroke, or cancer, WHR was significantly associated with the risk of incident CAD in both young (≤55 years) and older women, while other anthropometric measurements (including BMI, waist circumference, waist-length ratio, waist-to-sitting height ratio, WHtR, and Conicity index) were related to the risk of incident CAD, primarily among younger women only [28].

Tarastchuk *et al*. [29], investigated 308 patients who had undergone percutaneous coronary intervention (PCI), to determine the best anthropometric measurements of obesity for predicting MACE after PCI. Of the included measures (waist circumference, WHR, Conicity index and BMI), the authors found that only waist circumference was an independent predictor of MACE in men. Interestingly, BMI was not related to MACE but was in fact the least frequent abnormal anthropometric measure in patients with MACE [29].

A prospective study analyzed 250 patients with ST-Segment Elevation Myocardial Infarction (STEMI) treated with primary PCI. Different anthropometric measures were assessed, including body adiposity index (BAI), Conicity index, visceral adiposity index (VAI), waist circumference, WHR, and WHtR. The study investigated the relationship between MetS and obesity indices in predicting clinical severity and prognosis. Patients with MetS had higher rates of BMI ≥30 ${\mathrm{kg/m}}^{2}$ and central obesity (very high BAI, Conicity index >1.25/1.18; increased VAI, and WHtR ≥63/58). Among these indices, a WHtR ≥63/58 and a Conicity index >1.25/1.18 associated with a higher risk of in-hospital complications (OR 2.00, 95% CI 1.17–3.43; *p* = 0.011 and OR 3.30 95% CI 1.56–7.00; *p* = 0.002, respectively) [30].

Another study including 112 patients with myocardial infarction (MI) and 112 controls showed that most anthropometric measurements were significantly higher in MI patients. When the predictive ability of different indices was calculated, the umbilical WHR (c-index: 0.830), the umbilical WHtR (c-index: 0.788), the WHR (c-index: 0.796) and the Conicity index (c-index: 0.795) showed the highest values and best predictive performance. Surprisingly, BMI showed only a moderate c-index, suggesting that this measure may be inferior compared to WHR and Conicity index and a significant proportion of patients at risk of MI may be missed when using BMI [31].

In 2018, Nilsson *et al*. [32] studied 688 AMI patients younger than 80 years of age matched by sex and age with healthycontrols, and explored associations with basic anthropometric phenotypes. The predictive model that included hip circumference and weight was particularly efficient in discriminating men aged >65 years with MI from their controls. In men aged ≤65 years, the best combination was hip circumference, BMI, and height. In women >65 years, the best discriminatory model contained only the WHR, while in women ≤65 years, the best combination was hip circumference and BMI [32]. These data reinforce that there may be important sex-specific anthropometric phenotypes that need to be considered when predicting risk of CVD.

Finally, Rådholm *et al*. [33] followed 11,125 patients with T2DM and investigated WHtR as a predictor of risk of MACE. The risk of MACE was 16% higher per standard deviation increase in WHtR (HR 1.16; 95% CI 1.11–1.22), with WHtR (slightly) outperforming BMI and WHR [33]. Table 3 (Ref. [27, 28, 29, 30, 31, 32, 33]) summarizes the results of the included studies on other anthropometric measures and cardiovascular risk.

**Table 3.** **Studies included in the review about other anthropometric measurements**.

Study	Population	Sample size	Follow-up	Main outcomes
Khosravian *et al*. [27]	General population	1488	N/A	Predictive ability (as c-indexes) for metabolic syndrome of different anthropometric measurements:
				∙ Waist circumference = 0.743 (95% CI 0.71–0.77).
				∙ WHR = 0.602 (95% CI 0.57–0.63).
				∙ WHtR = 0.786 (95% CI 0.76–0.81).
				∙ Conicity index = 0.658 (95% CI 0.62–0.68).
Zhang *et al*. [28]	General population (females) free of CAD, stroke or cancer	67,334	2.5 years	Increased risk of developing CVD with the highest [tertile 3 *vs*. tertile 1]:
				∙ Waist circumference: RR 3.0 (95% CI 1.4–6.3).
				∙ WHR: RR 3.0 (95% CI 1.3–6.8).
				∙ Waist-to-sitting height ratio: RR 3.1 (95% CI 1.4–7.0).
				∙ Conicity index: RR 2.4 (95% CI 1.1–5.3).
				All models adjusted for age, smoking, alcohol consumption, physical activity, educational level, family income, menopause, hormone use, oral contraceptive use, recruitment season, and intake of soy fats, fibers and proteins.
Tarastchuk *et al*. [29]	Patients with CAD	308	6 months	Increased risk of MACE (any of the following: all-cause death, MI, cardiac surgery, reoperation, angina, or evidence of myocardial ischemia) in males with elevated waist circumference (>90 cm) (*p* = 0.0498) [*OR or HR not declared*].
Jelavic *et al*. [30]	Patients with STEMI	250	1 year	Increased risk of hospital complications with higher WHtR (≥63/58):
				∙ OR 2.00 (95% CI 1.17–3.43)
				Increased risk of hospital complications with higher Conicity index (>1.25/1.18):
				∙ OR 3.30 (95% CI 1.56–7.00).
Martín Castellanos *et al*. [31]	General population (controls) and patients with MI (cases)	224	N/A	Predictive ability (as c-indexes) for MI of different anthropometric measurements:
				∙ Waist circumference = 0.734 (95% CI 0.668–0.800).
				∙ WHR = 0.796 (95% CI 0.737–0.855).
				∙ WHtR = 0.761 (95% CI 0.698–0.823).
				∙ Conicity index = 0.795 (95% CI 0.738–0.853).
Nilsson *et al*. [32]	General population (controls) and patients with MI (cases)	1376	N/A	Predictive ability (as c-indexes) for MI of different anthropometric measurements:
				∙ Males >65 years: model with hip circumference and weight (c-index = 0.82; 95% CI 0.78–0.86).
				∙ Males ≤65 years: model with hip circumference, BMI and height (c-index = 0.79; 95% CI 0.75–0.83).
				∙ Females >65 years: model with WHR (c-index = 0.67; 95% CI 0.61–0.74).
				∙ Females ≤65 years: model with hip circumference and BMI (c-index = 0.68; 95% CI 0.58–0.76).
Rådholm *et al*. [33]	Patients with diabetes mellitus	11,125	9 years	Increased risk of MACE (any of the following: cardiovascular death, non-fatal MI, or non-fatal stroke) with higher WHtR:
				∙ HR 1.16 (95% CI 1.11–1.22) [per each standard deviation of increase].
				∙ HR 1.44 (95% CI 1.29–1.61) [tertile 3 *vs*. tertile1]
				Adjusted for age, sex, smoking, region, randomized intervention to lowering blood pressure and randomized intervention for glucose control.

RR, relative risk; OR, odds ratio; HR, hazard ratio; CI, confidence interval; CVD, cardiovascular disease; MI, myocardial infarction; MACE, major adverse cardiovascular events; WHR, waist-to-hip ratio; CAD, coronary artery disease; ACS, acute coronary syndrome; STEMI, ST-segment elevation myocardial infarction; BMI, body mass index; WHtR, waist-to-standing height ratio.

## 4. Usefulness of Skinfold Thickness, Waist-to-Hip Ratio and Other Anthropometric Measurements in Clinical Assessment

**Fig. 1.**
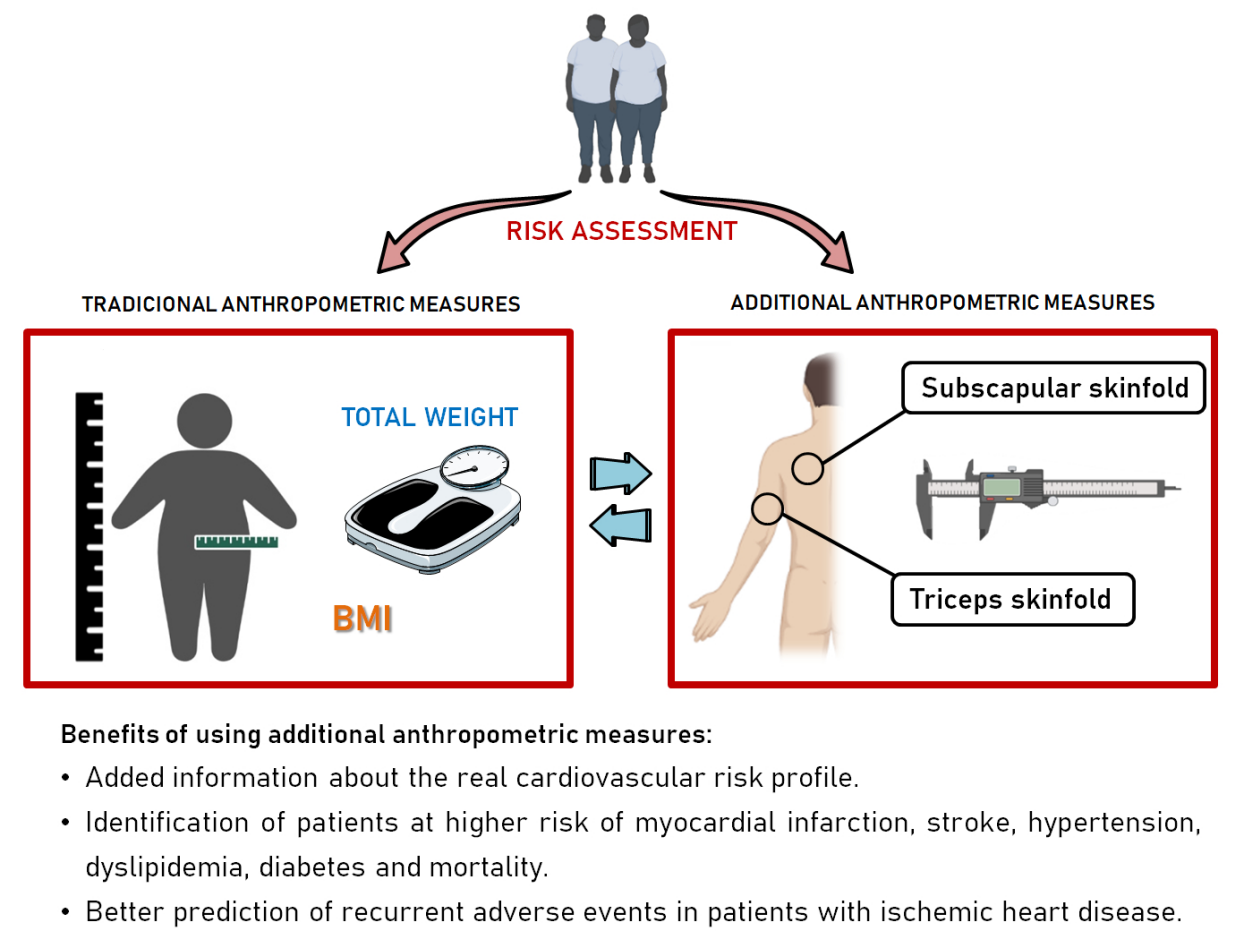
**Summary of the study findings**.

The diagnostic performance of the skinfold thickness method to detect obesity seems to be at least as useful as BMI [34]. As has been described before, two of the most commonly used skinfolds are TSF and the SSF, and several studies have shown that both could be useful for estimating cardiovascular risk in the general population and aid in the risk stratification process in patients without previous CVD (Fig. 1).

Some authors also argue that WHR may have a better predictive ability than BMI for mortality and incident CVD [35]. Indeed, in the study by Lee *et al*. [22], the highest mortality rate occurred in patients with the lowest BMI. This seems to support the ‘obesity paradox’, for which it has been reported a high BMI could associate with cardiovascular protection in some specific clinical contexts [36, 37]. However, the obesity paradox may simply be an outcome of selection bias in high-risk patient groups [38]. In addition, WHR is less influenced by muscle and bone mass and may therefore have certain advantages over BMI. Given the relationship of WHR with visceral adiposity, an increase in WHR implies a clear higher cardiovascular risk, whereas relying solely on BMI may underestimate the importance of obesity as a risk factor for CVD in people with some chronic conditions.

Similarly, the Conicity index has been demonstrated to predict several CVDs in the general population and has been related to an increase in metabolic and cardiovascular risk [39]. Previous studies have shown an association of the Conicity index with the development of diabetes and hypertension [40], as well as a good ability to estimate 10-year cardiovascular risk [41]. Based on the available evidence to date, it is possible to summarise that SSF, TSF, WHR and Conicity index predict cardiovascular events in the general population and patients without CVD (**Supplementary Table 1**).

Additionally, the parameters described above are not only interesting for evaluating the development of *de novo *CVD but also have utility in patients with prevalent CVD and cardiovascular risk factors. This emphasizes the importance of regular and routine assessment to identifying patients at elevated cardiovascular risk, thus preventing the occurrence of worse clinical outcomes and additional comorbidities.

In the case of skinfolds, a cross-sectional study including 3360 participants aged >60 years demonstrated that patients with AMI had a significantly higher SSF, among other anthropometric indices, compared to patients who did not suffer from AMI [42]. In another study carried out in Nigeria among rural and urban populations, the majority of anthropometric measurements including TSF, SSF, and the sum of five skinfold thicknesses (biceps, TSF, SSF, superior iliac and abdominal) were significantly higher in the urban population, in both men and women, and this population had a higher prevalence of various cardiovascular risk factors [43]. In a small study that sought to determine the prevalence of fatty liver disease in relation to different parameters in patients with familial hyperlipidaemia, several anthropometric parameters were correlated with the stages of fatty liver disease, with the SSF showing the strongest association compared to other skinfolds [44].

Similarly, a study in an Italian population showed that, among several adiposity indices, SSF was the best predictor of lower concentrations of insulin-like growth factor-binding protein (IGFBP)-1, an insulin-like hormone which plays an important role in child growth and continues having anabolic effects in adults. Therefore, simple measures of body adiposity, such as SSF, may represent an additional tool to improve phenotypic profiles associated with the pathogenetic mechanisms of clustering of cardiovascular risk factors in adults [45].

Regarding WHR in patients with previous CVD, a systematic review concluded that AMI is strongly associated with an increased WHR, with a stronger association among women [46]. Although most of the evidence regarding this association derives from developed countries, a case-control study showed that even in low-income and middle-income countries, abdominal obesity estimated by the WHR was an important risk factor presented in patients with AMI [47], and this is consistent in other regions of the world such as Latin America [48]. Even in certain populations such as patients with chronic kidney disease or diabetes, WHR but not BMI has been associated with cardiovascular events [49, 50].

Despite the availability of several anthropometric measurement tools with good cardiovascular risk prediction, they have typically been underused in clinical practice. There may be some benefit in measures collating several variables, such as the Conicity index described by Valdez *et al*. [39] in 1993, which is used to assess the degree of abdominal adiposity. However, the results so far are controversial, which highlights the need for more studies in this regard [51]. Moreover, anthropometric measurements can be useful to obtain clinical information beyond adiposity. For example, several studies revealed that a low hip circumference, a reflection of small gluteal muscles, had a negative association with MI, which could suggest an association between MI and sarcopenia, maybe in relation to physical inactivity and malnutrition [52, 53, 54, 55]. Thus, the application of these less traditional parameters, in conjunction or instead of BMI, could be useful in the clinical assessment of patients with and without CVD.

### 4.1 How to Correctly Assess Anthropometry in Clinical Practice?

As described, it is important to consider other parameters beyond the more conventional measures (mainly total weight and BMI). However, for these anthropometric measurements to be correctly interpreted and applied in clinical practice, they must be measured rigorously using consistent and standardized methods. This is particularly important for skinfolds, diameters, and perimeters, which should be based on the normative body of reference in Kinanthropometry, the International Society for the Advancement of Kinanthropometry (ISAK), which has developed international standards for anthropometric assessment and an international anthropometry accreditation scheme [9, 56].

Although the use of anthropometric measurements according to the requirements of this society has been applied mainly for the study of athletes and is little explored in clinical practice, the present study shows that it might also be useful for cardiovascular risk estimation and prediction of future adverse events. Specifically, it could be especially interesting for outpatients in primary prevention programs or patients included in comprehensive cardiac rehabilitation, since these are involved in programs in which nutrition and exercise are carefully controlled, and more frequents reviews and follow-ups are performed. In this way, and given the relationship exposed in this work between different anthropometric measures and cardiovascular risk, we think we should move forwards considering using these techniques in the general population and particularly in patients with CVD, to allow a more adequate assessment, and evaluate if the therapies, treatments, and lifestyles modifications are producing optimal results in such patients. Nevertheless, the personnel in charge of this process must be adequately trained and perform such anthropometric measures according to an international consensus.

### 4.2 Limitations

There are some limitations in relation to this work. First, there is contradictory information in the scientific literature, as has been discussed in this review. Some studies have shown the relationship of certain anthropometric parameters with cardiovascular risk, while others did not. There is also a lack of sufficient comparisons between BMI and other anthropometric parameters, including SSF and TSF, which hinder our ability to make strong conclusions of superiority. There is substantial heterogeneity in the specific parameters and cut-off points used for many anthropometric measures. Moreover, the high heterogeneity of the anthropometric parameters used in the different studies, as well as the clinical outcomes evaluated, hindered performing a meta-analysis or showing pooled results. This highlights the importance of performing further studies in this field and our observations should be interpreted with caution at this time.

## 5. Conclusions

Different anthropometric parameters are useful for predicting cardiovascular risk in the general population, and recurrent adverse events in patients with previous CVD. In patients from the general population, higher SSF and TSF were associated with diabetes, hypertension, hypercholesterolemia, all-cause death and cardiovascular death. In addition, SSF was associated with higher risk of CAD and stroke.WHR and Conicity index identified patients from the general population with higher risk of CVD, and MI, as well as patients with previous CVD at higher risk of hospital complications and recurrent MI. These less used anthropometric measures such as SSF, TSF, WHR and the Conicity index, might improve risk stratification and evaluation of therapeutic interventions compared to BMI.

## Supplementary Material

Supplementary material associated with this article can be found, in the online version, at https://doi.org/10.31083/j.rcm2312414.
