# Supplementary material for: Anthropometric Measures and Risk of Cardiovascular Disease: Is there an Opportunity for Non-Traditional Anthropometric Assessment? A Review
Source: Rev Cardiovasc Med. 2022 Dec 20;23(12):414. doi: 10.31083/j.rcm2312414 (PMC11270468; doi:10.31083/j.rcm2312414)
Supplement: Supplementary file 1 [file 2153-8174-23-12-414-s1.docx]

# SUPPLEMENTARY MATERIAL

# Supplementary Table 1. Studies included in the review about non-traditional anthropometric measures and risk of cardiovascular disease in the general population or patients without previous cardiovascular disease.

| **Study** | **Population** | **Sample size** | **Follow-up** | **Main outcomes** |
| --- | --- | --- | --- | --- |
| Donahue et al. (1) | Patients free of previous CVD | 7,692 | 12 years | Increased incidence of CAD in patients with the highest tertile of the SSF (80 patients per 1000, p<0.001).  Increased risk of developing CAD with higher SSF fold:   - RR 2.2 (95% CI 1.8-2.8) [tertile 3 *vs*. tertile 1, adjusted by age]. - RR 1.5 (95% CI 1.1-2.1) [tertile 3 *vs*. tertile 1, adjusted by risk factors]. |
| Kannel et al. (2) | General population | 5,209 | 24 years | Increased risk of developing CVD with higher SSF (quintile 5 *vs*. quintile 1).   - CAD: RR 1.8 (p<0.001) (males); RR 1.8 (p<0.001) (females). - Stroke: RR 1.7 (p<0.01) (females). - Any CVD: RR 1.4 (p<0.001) (males); RR 1.7 (p<0.001) (females). - CAD-related mortality: RR 1.4 (p<0.001) (males); RR 2.0 (p<0.001) (females). - Cardiovascular mortality: RR 1.4 (p<0.001) (males); RR 1.5 (p<0.001) (females). |
| Yarnell et al.(3) | Patients (males) free of previous CVD | 2,512 | 14 years | Increased risk of developing CAD with higher SSF:   - OR 1.23 (95% CI 1.04-1.45) [per standard deviation of increase, adjusted for BMI]. - RR 1.90 (95% CI 1.30-2.80) [quintile 5 *vs*. quintile 1, adjusted for age, smoking habit and social class]. |
| Iso et al. (4) | General population | 10,582 | 17 years | Increased risk of non-embolic ischemic stroke in non-hypertensive diabetic subjects with higher SSF (RR 4.9; 95% CI 2.5-9.5) and TSF (RR 3.6; 95% CI 1.7 -7.4). |
| Tane et al. (5) | Patients (males) free of previous CVD | 9,151 | 23 years | Higher overall mortality rate from CAD or stroke in the fourth quartile of the SSF.  Increased risk of mortality from CAD with higher SSF:   - HR 1.13 (95% CI 1.06-1.20) [adjusted for age]. - HR 1.06 (95% CI 1.00-1.13) [adjusted for age and hypertension].   Increased risk of mortality from stroke with higher SSF:   - HR 1.12 (1.01-1.25) [adjusted for age]. |
| Chei et al. (6) | General population from two communities | 5,617 | 10 years | Increased risk of developing hypertension in females from one of the communities with the highest SSF:   - OR 1.60 (95% CI 1.04-2.46) [tertile 3 *vs*. tertile 1].   Increased risk of developing diabetes in females from both communities with higher SSF:   - OR 2.06 (95% CI 1.05-4.04) [tertile 3 *vs*. tertile 1]. - OR 3.58 (95% CI 1.33-9.64) [tertile 3 *vs*. tertile 1]. |
| Patel et al. (7) | General population | 8,892 | N/A | Moderate predictive ability of the sum of the TSF to SSF in patients between 20 and 60 years of age for:   - Hypercholesterolemia (c-index = 0.617 [males]; 0.689 [females]). - Diabetes (c-index = 0.764 [males]; 0.774 [females]). - Hypertension (c-index = 0.693 [males]; 0.768 [females]). |
| Loh et al. (8) | Patients (males) free of previous CVD | 870 | 27.7 years | Increased risk of mortality with the lowest iliac skinfold:   - All-cause mortality: HR 0.77 (95% CI 0.66-0.90). - Arteriovascular mortality: HR 0.75 (95% CI 0.58-0.97). - Infection mortality: HR 0.63 (95% CI 0.42-0.94). |
| Ruiz-Alejos et al. (9) | General population | 988 | 7.6 years | Increased risk of diabetes in patients with higher SSF: RR 5.04 (95% CI 1.85-13.73).  Increased risk of hypertension in patients with higher SSF: RR 2.15 (95% CI 1.30-3.55).  *Both models adjusted for age, sex, education, assets index, smoking, excessive alcohol consumption, population group, level of physical activity and BMI. |
| Liu et al. (10) | General population | 16,402 | 11.81 years | Lower risk for all-cause and cardiovascular mortality in participants in the highest quartile of SSF.   - All-cause mortality: HR 0.71 (95% CI 0.57-0.89) [quartile 4 *vs*. quartile 1]. - Cardiovascular mortality: HR 0.44 (95% CI 0.23-0.83) [quartile 4 *vs*. quartile 1].   *Both models for age, gender, race, education level, marital status, smoking, alcohol consumption, BMI, systolic blood pressure, estimated glomerular filtration rate, high-density lipoprotein cholesterol, total cholesterol, C-reactive protein, comorbidities, and medication use. |
| Li et al. (11) | General population | 62,160 | 119 months | Lower risk for all-cause and cardiovascular mortality in participants in the highest quartile of TSF.   - All-cause mortality: HR 0.64 (95% CI 0.54-0.76) [quartile 4 *vs*. quartile 1]. - Cardiovascular mortality: HR 0.54 (95% CI 0.36-0.79) [quartile 4 *vs*. quartile 1].   *Both models for age, gender, race, waist circumference, education level, marital status, smoking, BMI, estimated glomerular filtration rate, high-density lipoprotein cholesterol, total cholesterol, and comorbidities. |
| Yusuf et al. (12) | General population (controls) and patients with a first episode of MI (cases) | 27,098 | N/A | Increased risk of MI with higher WHR:   - OR 1.37 (95% CI 1.33-1.40) [for each standard deviation, adjusted for age, sex, region, BMI and height]. - OR 1.33 (95% CI 1.16-1.53) [quintile 5 *vs*. quintile 1, adjusted for age, sex, region, BMI, height, smoking, apolipoproteins, hypertension, diabetes, diet, physical activity, alcohol, and psychosocial variables]. |
| Myint et al. (13) | General population | 15,062 | 11.7 years | Increased risk of mortality with higher WHR:   - HR 1.42 (95% CI 1.14-1.78) (in females).   Increased risk of developing CVD with higher WHR:   - Males: HR 1.17 (95% CI 1.01-1.36). - Females: HR 1.36 (95% CI 1.16-1.58).   All models adjusted for age, smoking, alcohol consumption, physical activity, social class, education, blood pressure, cholesterol, diabetes, stroke, MI, cancer, % of body fat and BMI. |
| Egeland et al. (14) | General population | 140,790 | 11.5 years | Increased risk of MI with higher WHR:   - Males <60 years: HR 1.22 (95% CI 1.07-1.40)*. - Females <60 years: HR 1.76 (95% CI 1.37-2.25)*.   *Both models for the two highest quintiles and adjusted for age, smoking, BMI, systolic blood pressure, diabetes, and total cholesterol-HDL ratio. |
| Khosravian et al. (15) | General population | 1,488 | N/A | Predictive ability (as c-indexes) for metabolic syndrome of different anthropometric measurements:   - Waist circumference = 0.743 (95% CI 0.71-0.77). - WHR = 0.602 (95% CI 0.57-0.63). - WHtR= 0.786 (95% CI 0.76-0.81). - Conicity index = 0.658 (95% CI 0.62-0.68). |
| Zhang et al. (16) | General population (females) free of CAD, stroke or cancer | 67,334 | 2.5 years | Increased risk of developing CVD with the highest [tertile 3 *vs*. tertile 1]:   - Waist circumference: RR 3.0 (95% CI 1.4-6.3). - WHR: RR 3.0 (95% CI 1.3-6.8). - Waist-to-sitting height ratio: RR 3.1 (95% CI 1.4-7.0). - Conicity index: RR 2.4 (95% CI 1.1-5.3).   All models adjusted for age, smoking, alcohol consumption, physical activity, educational level, family income, menopause, hormone use, oral contraceptive use, recruitment season, and intake of soy fats, fibers and proteins. |
| Martín Castellanos et al. (17) | General population (controls) and patients with MI (cases) | 224 | N/A | Predictive ability (as c-indexes) for MI of different anthropometric measurements:   - Waist circumference = 0.734 (95% CI 0.668-0.800). - WHR = 0.796 (95% CI 0.737-0.855). - WHtR= 0.761 (95% CI 0.698-0.823). - Conicity index = 0.795 (95% CI 0.738-0.853). |
| Nilsson et al. (18) | General population (controls) and patients with MI (cases) | 1,376 | N/A | Predictive ability (as c-indexes) for MI of different anthropometric measurements:   - Males >65 years: model with hip circumference and weight (c-index = 0.82; 95% CI 0.78-0.86). - Males ≤65 years: model with hip circumference, BMI and height (c-index = 0.79; 95% CI 0.75-0.83). - Females >65 years: model with WHR (c-index = 0.67; 95% CI 0.61-0.74). - Females ≤65 years: model with hip circumference and BMI (c-index = 0.68; 95% CI 0.58-0.76). |
| BMI = body mass index; CAD = coronary artery disease; CI = confidence interval; CVD = cardiovascular disease; HR = hazard ratio; MI = myocardial infarction; OR = odds ratio; RR = relative risk; SSF = subscapular skinfold; TSF = triceps skinfold; WHR = waist-to-hip ratio; WHtR = waist-to-standing height ratio. | | | | |

**References**

1. Donahue RP, Abbott RD, Bloom E, Reed DM, Yano K. Central obesity and coronary heart disease in men. Lancet. 1987;1(8537):821-4.

2. Kannel WB, Cupples LA, Ramaswami R, Stokes J, 3rd, Kreger BE, Higgins M. Regional obesity and risk of cardiovascular disease; the Framingham Study. J Clin Epidemiol. 1991;44(2):183-90.

3. Yarnell JW, Patterson CC, Thomas HF, Sweetnam PM. Central obesity: predictive value of skinfold measurements for subsequent ischaemic heart disease at 14 years follow-up in the Caerphilly Study. Int J Obes Relat Metab Disord. 2001;25(10):1546-9.

4. Iso H, Imano H, Kitamura A, Sato S, Naito Y, Tanigawa T, et al. Type 2 diabetes and risk of non-embolic ischaemic stroke in Japanese men and women. Diabetologia. 2004;47(12):2137-44.

5. Tanne D, Medalie JH, Goldbourt U. Body fat distribution and long-term risk of stroke mortality. Stroke. 2005;36(5):1021-5.

6. Chei CL, Iso H, Yamagishi K, Tanigawa T, Cui R, Imano H, et al. Body fat distribution and the risk of hypertension and diabetes among Japanese men and women. Hypertens Res. 2008;31(5):851-7.

7. Patel SA, Deepa M, Shivashankar R, Ali MK, Kapoor D, Gupta R, et al. Comparison of multiple obesity indices for cardiovascular disease risk classification in South Asian adults: The CARRS Study. PLoS One. 2017;12(4):e0174251.

8. Loh WJ, Johnston DG, Oliver N, Godsland IF. Skinfold thickness measurements and mortality in white males during 27.7 years of follow-up. Int J Obes (Lond). 2018;42(11):1939-45.

9. Ruiz-Alejos A, Carrillo-Larco RM, Miranda JJ, Gilman RH, Smeeth L, Bernabé-Ortiz A. Skinfold thickness and the incidence of type 2 diabetes mellitus and hypertension: an analysis of the PERU MIGRANT study. Public Health Nutr. 2020;23(1):63-71.

10. Liu XC, Liu L, Yu YL, Huang JY, Chen CL, Lo K, et al. The Association of Subscapular Skinfold with All-Cause, Cardiovascular and Cerebrovascular Mortality. Risk Manag Healthc Policy. 2020;13:955-63.

11. Li W, Yin H, Chen Y, Liu Q, Wang Y, Qiu D, et al. Associations Between Adult Triceps Skinfold Thickness and All-Cause, Cardiovascular and Cerebrovascular Mortality in NHANES 1999-2010: A Retrospective National Study. Front Cardiovasc Med. 2022;9:858994.

12. Yusuf S, Hawken S, Ounpuu S, Bautista L, Franzosi MG, Commerford P, et al. Obesity and the risk of myocardial infarction in 27,000 participants from 52 countries: a case-control study. Lancet. 2005;366(9497):1640-9.

13. Myint PK, Kwok CS, Luben RN, Wareham NJ, Khaw KT. Body fat percentage, body mass index and waist-to-hip ratio as predictors of mortality and cardiovascular disease. Heart. 2014;100(20):1613-9.

14. Egeland GM, Igland J, Vollset SE, Sulo G, Eide GE, Tell GS. High population attributable fractions of myocardial infarction associated with waist-hip ratio. Obesity (Silver Spring). 2016;24(5):1162-9.

15. Khosravian S, Bayani MA, Hosseini SR, Bijani A, Mouodi S, Ghadimi R. Comparison of anthropometric indices for predicting the risk of metabolic syndrome in older adults. Rom J Intern Med. 2021;59(1):43-9.

16. Zhang X, Shu XO, Gao YT, Yang G, Matthews CE, Li Q, et al. Anthropometric predictors of coronary heart disease in Chinese women. Int J Obes Relat Metab Disord. 2004;28(6):734-40.

17. Martín Castellanos Á, Cabañas Armesilla MD, Barca Durán FJ, Martín Castellanos P, Gómez Barrado JJ. Obesity and risk of myocardial infarction in a sample of European males. Waist to-hip-ratio presents information bias of the real risk of abdominal obesity. Nutr Hosp. 2017;34(1):88-95.

18. Nilsson G, Hedberg P, Leppert J, Ohrvik J. Basic Anthropometric Measures in Acute Myocardial Infarction Patients and Individually Sex- and Age-Matched Controls from the General Population. J Obes. 2018;2018:3839482.
